# The Prevalence of Reproductive Tract Infections in a Chinese Internal Migrant Population, and Its Correlation with Knowledge, Attitude, and Practices: A Cross-Sectional Study

**DOI:** 10.3390/ijerph16040655

**Published:** 2019-02-22

**Authors:** Shuangfei Xu, Chuanning Yu, Ying Zhou, Junqing Wu, Tieling Bai, Junxian Zhang, Yuyan Li

**Affiliations:** 1 Key Lab of Reproduction Regulation of National Population and Family Planning Commission, Shanghai Institute of Planned Parenthood Research, Institute of Reproductive Development, Fudan University, Shanghai 210035, China; 15211150007@fudan.edu.cn (S.X.); yingzhou2012@163.com (Y.Z.); wujq1688@163.com (J.W.); 2 School of Public Health, Fudan University, Shanghai 200032, China; 3 Zhejiang Provincial Center for Disease Control and Prevention, Hangzhou 310051, China; 4 Longhua District Center for Chronic Disease Control (Mental Health Center), Shenzhen 518110, China; yu.chuanning@icloud.com; 5 Family Planning and Reproductive Health Instructing Center of Ningxia Hui Autonomous Region, Yinchuan 750000, China; luxn001@163.com; 6 Family Planning Publicity and Education Technical Advising Center of Urumchi, Urumchi 830092, China; junxianzhang@sina.cn

**Keywords:** reproductive tract infection, knowledge, attitude, and practices (K.A.P), internal migrant population, China

## Abstract

*Objective*: This study was designed to assess the prevalence of reproductive tract infections (RTIs) among an internal-migrant population of reproductive age in China. We also analyzed the knowledge, attitude, and practices related to these infections. *Methods*: A cross-sectional study using the quota-sampling method was conducted in three cities from March 2016 to February 2017. A total of 3320 participants (40.7% men and 59.3% women) were enrolled in the study, of whom, 1124, 1015, and 1181 were from Yinchuan, Urumchi, and Shanghai, respectively. Data on the included subjects were collected using a self-administered questionnaire. *Results*: We found that 3.2% and 22.6% of all subjects lacked knowledge regarding the identification and prevention of RTIs, respectively. More than 80% of the participants approved of developing RTI surveillance and taking RTI-related courses. While 45.1% of the respondents changed their underwear every 2–3 days, 49.0% cleaned their genitals daily, and 34.9% reported taking a bath daily. Among contraceptive users, 47.4% and 29.7% used condoms and IUDs (intrauterine devices), respectively. Overall, 48.2% of the participants had laboratory-confirmed sexually transmitted infections, and 19.7% of the female participants had endogenous infections. After controlling the socio-demographic variables, participants who cleaned their genitals and took a bath less frequently, as well as used condoms or pills had a lower prevalence of sexually transmitted infections, while those who were unwilling to take RTI-related courses and changed their underwear less frequently were more likely to contract sexually transmitted infections. While women who were unwilling to take RTI-related courses had a lower prevalence of endogenous infections, those with more children had a higher prevalence. *Conclusion*: The status of RTIs among the internal-migrant population of reproductive-age is not optimistic and is related to multiple factors. We believe this study will contribute to improving the knowledge, attitude, and practices related to RTIs.

## 1. Introduction

It is estimated that approximately one million new cases of curable sexually transmitted infections (STIs), such as chlamydia, gonorrhea, trichomoniasis, and syphilis, occur daily worldwide in individuals aged 15–49 years [1]. In 2012, the estimated global prevalence of chlamydia, gonorrhea, trichomoniasis, and syphilis among women of reproductive-age was 4.2%, 0.8%, 5.0%, and 0.5%, respectively, while that among men was 2.7%, 0.6%, 0.6%, and 0.5%, respectively [1]. In the United States, the prevalence of bacterial vaginosis (BV) among women was 29.2% [2], and nearly 75% of adult women have contracted vulvovaginal candidiasis (VVC) at least once in their lifetime (on rare occasions, men can also get genital candidiasis) [3]. In the UK, BV was twice as prevalent as VVC and affected a third of the women at least once in their lifetime [4].

In China, in 2010, the self-reported rate of sexually transmitted diseases (STDs) was 5.4% and 5.3% in men and women, respectively [5]. Notably, population mobility was an influencing factor associated with the rising incidence of STDs [6]. In 2015, the internal migrant population of 247 million accounted for 17.9% of the total Chinese population. The majority (76.6%) of these migrants are aged between 15 to 59 years and are characterized by active sexual lives, low educational level, and poor knowledge of reproductive health. Previous studies have reported that 30.3–62.0% of the internal migrant women population were diagnosed with laboratory-confirmed reproductive tract infections (RTIs), which included BV (11.6–22.2%), VVC (4.9–28.1%), trichomoniasis (2.1–12.7%), gonorrhea (0.12–1.6%), syphilis (0.18–0.2%), and cervicitis with no laboratory-classification (21.6–59.8%) [7,8,9,10,11]. However, studies on RTIs among the male internal migrant population are quite rare, though a few of them have reported that 14.9–36.2% of this population have urogenital tract infections (UTIs) [12,13].

In this study, we aimed to describe the status of RTIs (including STIs) among the reproductive-aged internal-migrant population in China. Meanwhile, knowledge, attitude, and practice (K.A.P) reflects respondents’ understanding of any given topic, their feelings and preconceived ideas towards it, and ways in which they demonstrate their knowledge and attitude through their actions [14]. Understanding the levels of K.A.P will enable a more efficient process of awareness creation, as it will allow the program to be tailored more appropriately to the needs of the internal migrants. Thus, we also portrayed RTI-related K.A.P and analyzed the correlation between the K.A.P and RTIs.

## 2. Materials and Methods

### 2.1. Definition

RTIs are caused by bacterial, parasitic, and viral pathogens [15], and include three types of infections: (1) STIs such as chlamydia, gonorrhea, and human immunodeficiency virus (HIV); (2) endogenous infections, such as BV and VVC, that are caused by the overgrowth of organisms normally present in the genital tract of healthy people, and (3) iatrogenic infections, caused by unsafe abortions or poor delivery practices [16]. In this study, the term “RTIs” refers to only STIs and endogenous infections.

### 2.2. Sample Size

The sample size was calculated based on the following formula:

(1) $$N=\mathrm{deff}\times Z_{\alpha }^{2}\times p\times \left(1-p\right)/\delta^{2}$$

where *N* is the parameter to be calculated and is the sample size in terms of number of internal migrant population to be selected; deff is the sample design effect, assumed to be 2.0 (default value); $Z_{\alpha }^{2}$ is the statistic that defines the level of confidence desired. The z-statistic to use should be 1.96 for the 95% confidence interval; *p* is an estimate of a key indicator to be measured by the study, and here, according to a pilot study carried out in Shanghai with 60 participants, *p* equals 45%; δ is the margin of error to be attained. It is recommended to set the level of precision at 10% of *p*.

Based on the above calculation, a minimal sample size of 940 was required. Therefore, for all the 3 survey sites together, the total sample size was 2820 (940 × 3).

### 2.3. Sampling Strategy and Study Population

This study was conducted in the cities of Yinchuan, Urumchi, and Shanghai. In each city, two districts were randomly selected. The sampling cities had to meet the following two criteria. First, the concentration of migrant population was essential, which meant that the selected city (1) possessed intensive industries, concentrated areas in the city and town, and convenient transportation, which are attractive to the internal migrant population; and (2) harbored a sufficiently-large, eligible internal migrant population. Second, the selected areas should have a good foundation for family planning, which indicated: (1) The local government supports the program and pays attention to immigrants’ reproductive health; (2) the Department of Service and Management of Migrant Population cooperates with the survey and actively participates in the study; and (3) the selected district is equipped with adequate professional public health agencies (such as community health centers, family planning stations, health education centers, and maternity and child care centers) and medical technical personnel.

Shanghai is the largest city in East China. Yinchuan and Urumchi, as the provincial capitals of Ningxia Hui Autonomous Region and Sinkiang Autonomous Region, respectively, are the main inflowing regions for internal migrants in Northwest China. During the preparation stage, we found that there were only a few studies on the sexual and reproductive health (SRH) of internal migrants in Northwest China. Therefore, Shanghai, Yinchuan, and Urumchi were chosen for this study.

The method of quota-sampling was employed to recruit a composite sample which was approximately proportional to the distribution of internal migrants’ occupations: (1) Laborers, who work in manufacturing (textile, plastic products, metal products, and electronic equipment), construction (building, decoration), transport, storage, and expressage; (2) service workers, who have jobs in accommodation, catering, sauna, and entertainment; (3) white collar workers, who are employed in education, accounting, and administrative management; and (4) others, who work in wholesale, retail trades, and so on. The inclusion criteria for eligible respondents were: (1) 15–49 years of age; (2) had resided in the current cities for at least three months; (3) were not registered as permanent residents; and (4) volunteered to participate in this study.

A total of 3771 internal migrants of reproductive age were recruited from March 2016 to February 2017, of which 1174 came from Yinchuan, 1253 from Urumchi, and the remaining 1344 from Shanghai. Of the recruited subjects, 88.0% (3320/3771; 1124 from Yinchuan, 1015 from Urumchi, and 1181 from Shanghai) participated in the whole investigation, including the questionnaire, clinical examination, and laboratory testing.

### 2.4. Measures

#### 2.4.1. Socio-Demographic Characteristics

Conceptual definition: A group of traits related to the size, territorial distribution, and composition of the population, changes therein, and the components of such changes [17].

Operational definition: Variables to be assessed are collected from self-administered questionnaires, include area, age, sex, educational level, occupation, marital status, family per capita monthly income (CNY), type of household registration (hukou), duration of staying at the residence per year (Table S1).

#### 2.4.2. RTI-Related K.A.P

Conceptual definition: A “knowledge, attitudes, and practices (K.A.P)” survey is a representative study of a specific population that aims to collect data on what is known, believed and done in relation to a particular topic [18]. In this regard, RTI-related K.A.P reflects respondents’ understanding of RTIs, their feelings and preconceived ideas about it, and ways in which they demonstrate their knowledge and attitude through their actions [14].

Operational definition: Variables to be assessed are collected from self-administered questionnaires, included knowledge of identification and prevention of RTIs, attitude towards the development of RTI general investigation and willingness to take RTI-related educational courses, practices of personal hygiene (frequency of changing underwear, cleaning one’s genitals, and taking a bath, bath methods, and whether or not to have sex during menstruation), whether or not having RTI symptoms in last month, number of sexual partners, number of children (above variable only for the female internal migrant population), and contraceptive methods used (Table S2). The questionnaire for testing knowledge of RTI identification included 21 questions (such as “whether BV is an RTI or not”, and “whether frequent micturition, urgent urination, and odynuria are symptoms of RTI or not”), while that for testing knowledge of RTI prevention included 7 questions (such as “whether using a condom correctly and consistently is a preventive measure for RTIs or not”). Each question had three alternative answers: “yes”, “no”, and “do not know”, and only one answer was correct. The respondent scored 1 and 0 points for every right and wrong answer, respectively. The sum of the scores from the two tests were then converted to the centesimal system to obtain the final scores for knowledge of RTI identification and prevention, which were used for all further analyses.

#### 2.4.3. Prevalence of RTIs

Conceptual definition: The proportion of the investigated population that has laboratory-confirmed STIs or endogenous infections.

Operational definition: Variables to be assessed are collected from laboratory diagnosis results, which included STIs (trichomonal vaginitis (TV), chlamydia trachomatis (CT), neisseria gonorrhoeae (NGH), ureaplasma urealyticum (UU), syphilis, and HIV), as well as endogenous infections (BV and VVC) (Table S3).

Laboratory examination items and diagnostic methods:

Fasting blood, vaginal and cervical discharge (for female) and urethral discharge (for male) were collected.

(1) Trichomoniasis vaginitis

Typical motile trichomonads were identified in a saline wet mount of female vaginal discharge or male urethral discharge.

(2) Chlamydia trachomatis

Nucleic acid amplification test (NAAT) for female endometrial swabs or male urethral swabs: polymerase chain reaction (PCR) was selected in this study.

(3) Neisseria gonorrhoeae

NAAT for female endometrial swabs or male urethral swabs: PCR was selected in this study.

(4) Ureaplasma urealyticum

NAAT for female endometrial swabs or male urethral swabs: PCR was selected in this study.

(5) Human immunodeficiency virus (HIV)

If an enzyme linked immunosorbent assay (ELSA) test for HIV antibody was positive, a western blotting (WB) test was selected in this study.

(6) Syphilis

Serologic detection of syphilis:

A. If a serologic test of non-treponema pallidum antigen was positive, a rapid plasma reaction actor (RPR) was selected in this study;

B. If a serologic test of non-treponema pallidum antigen was positive, a passive particle agglutination test for treponema pallidum (TPPA) was selected.

(7) Bacterial vaginosis

Meet at least three of the four Amsel diagnostic criteria:

A. Homogeneous white to off-white sticky discharge;

B. Elevated vaginal pH (>4.5);

C. Fishy odor of vaginal discharge after addition of 1–2 drops of 10% potassium hydroxide (KOH);

D. The presence of clue cells in saline wet mount or gram stain of vaginal discharge.

(8) Vulvovaginal candidiasis

Microscopic identification of yeast cells or hyphae in 10% KOH in wet-mount preparations of vaginal discharge.

### 2.5. Age Standardization

In consideration of the different age structures in the three cities, it is necessary to standardize the prevalence of RTIs before comparison. Therefore, data on the internal migrant population from the Sixth Nationwide Population Census was selected as the reference population (Table S4).

## 3. Ethics Statement

The study protocol was approved by the Research Ethics Committee of the Shanghai Institute of Planned Parenthood Research (code: PJ2014-20). Before data collection, the purpose and procedures of the study were explained to all the eligible participants. Verbal and written informed consents for privacy protection and information security were obtained from all the participants, including parents of the minors. They fully comprehended their privilege to quit the research at any time they wanted without any adverse consequences.

## 4. Results

### 4.1. Socio-Demographics Characteristics.

In total, 3320 eligible participants (men: 40.7% and women: 59.3%) were included in this study. Of the included subjects, 50% were between the ages of 25–34 years and more than 90% of them were married. While 76.4% came from rural areas, 76.9% had resided in the current residence for a whole year. The majority (42.4%) of the respondents finished junior high school, and approximately 50% of the families earned 3000–4999 (CNY) per capita monthly in the last year. Additionally, while 39.9% of the participants were laborers, 22.6% were service workers (sauna: 221/749 and entertainment: 241/749) (Table 1).

### 4.2. RTI-Related K.A.P

#### 4.2.1. RTI-Related Knowledge

Of all the participants, 3.2% and 22.6% scored 0 points on the RTI identification and RTI prevention knowledge questionnaires, respectively. The median scores for men were 57.1 on both the tests, while for women they were 61.9 (RTI identification) and 57.1 (RTI prevention). Based on the Wilcoxon test, the median score for the knowledge of RTI identification was higher than that for RTI prevention (S = 472,819, *p* < 0.0001). While women had a higher median score on the RTI identification knowledge (Z = −3.1, *p* = 0.0021), men had a higher median score on the RTI prevention knowledge (Z = 3.7, *p* = 0.0002) questionnaires.

#### 4.2.2. RTI-Related Attitude and Practices

As for RTI associated attitude, while 84.5% of the participants considered it essential to develop RTI surveillance, 3.1% thought it was unessential, and the remaining 12.4% were uncertain. The majority (96.1%) of the respondents were willing to take RTI-related educational courses while the remaining 3.9% were unwilling to. Moreover, explanation/counselling from doctors (63.2%), brochures (59.4%), and lectures (39.3%) were the top three preferred education methods (Table 2).

In terms of RTI-associated practices, while 45.1% of the participants changed their underwear every 2 or 3 days, 49.0% cleaned their genitals daily, 34.9% of them took a bath daily, and the majority of them (90.4%) preferred to shower. Meanwhile, approximately 80.2% of the participants denied having sex during menstruation and 24.9% reported at least one RTI symptom in the last month. While 28.0% of the participants did not adopt any contraceptive methods, 47.4% and 29.7% of the remaining used condoms and IUDs, respectively. Besides, 97.6% of the respondents had one sexual partner, 1.0% had two, 0.4% had more than three, and 1.0% denied to answer. While 39.2% of the women had one child, 29.7% of them had none (Table 2).

Besides, we found the distributions of RTI-related attitude and practices (whether or not willing to take RTI-related education courses, frequency of changing underwear, frequency of cleaning genitals, frequency of taking bath, bath methods, whether or not having RTI symptoms in the last month, contraceptive methods in use, number of sexual partners and number of children) were different in each age group (*p* < 0.05). Younger participants were more inclined to take RTI-related education courses and preferred to shower. Participants in the older age group; however, more frequently changed underwear, cleaned genitals, and took a bath. More of the older group had RTI-symptoms in the last month, used contraceptives, and had more sexual partners. They mostly adopted IUD and sterilization rather than condoms, which was more popular among the younger. In addition, older female participants had more children.

### 4.3. Laboratory-Confirmed RTIs

Overall, 48.2% of the study participants had laboratory-confirmed STIs, and 19.7% of the women had endogenous infections. Among those with STIs, 44.0% were men, and 51.0% were women. Moreover, the rate of UU infection, which was the most common, was 41.3% in men and 48.3% in women. Table 3 summarizes the information on laboratory-confirmed RTIs among participants in each city. Using the internal migrant population from the Sixth Nationwide Population Census as the reference population, the total age-adjusted rate of STIs was 47.8%, and that of endogenous infections was 17.6%. The age-adjusted rates of STIs in Yinchuan, Urumchi, and Shanghai were 40.9%, 46.2%, and 57.6%, respectively, that among men were 38.7%, 40.8%, and 49.3%, respectively, and those in women were 41.8%, 47.7%, and 71.0%, respectively. The age-adjusted rates of endogenous infections in Yinchuan, Urumchi, and Shanghai were 18.8%, 21.9%, and 11.4%, respectively (Table 4).

### 4.4. Univariable and Multivariable Analyses of RTIs

In the univariable analyses of RTIs, the following factors were associated with both STIs and endogenous infections: age, education attainment, occupation, belief in the necessity to develop RTI surveillance, willingness to take RTI-related education, whether or not having RTI symptoms in the last month, and contraceptive methods.

For STIs, other associated factors included region, sex, family per capita monthly income, type of household registration (hukou), the frequency of cleaning genitals, and frequency of taking a bath. Factors that were associated with endogenous infections included bath methods and the number of children (Table 5).

The socio-demographic characteristics and RTI-related K.A.P variables were used for the multivariable analyses of STIs and endogenous infections. Consequently, the occurrence of STIs was notably related with a willingness to take RTI-related educational courses, RTI symptoms, contraceptive methods, and the frequency of changing underwear, cleaning genitals, and taking a bath (*p* < 0.05). If study participants were unwilling to take RTI-related courses, the occurrence of STIs was more likely to increase (OR = 1.6, 95% CI: 1.1–2.3). Additionally, while STIs were more likely to occur in participants who changed their underwear less frequently, they were less likely to occur in those with a lower frequency of cleaning genitals and taking a bath. While for the study participants who had RTI symptoms in last month, the occurrence of STIs was more likely to increase (OR = 1.3, 95% CI: 1.1–1.6). The study respondents who used condoms or pills for contraception were less likely to have STIs than those who used sterilization (Table 6).

Multivariable analyses also revealed that whether participants were willing to take RTI-related courses or not, RTI symptoms and the number of children were significant associated with endogenous infections (*p* < 0.05). The prevalence of these infections was, however, less likely to increase in participants who were unwilling to take RTI-related courses (OR = 0.4, 95% CI: 0.2–0.9). Additionally, the respondents who had RTI symptoms in last month were more likely to have endogenous infections (OR = 3.2, 95% CI: 2.5–4.0). Furthermore, the probability of having endogenous infections was significantly higher in participants who had more than one child (Table 6).

## 5. Discussion

This population-based study reveals poor reproductive health among the young internal migrant population in three Chinese cities. While 44.0% and 51.0% of the male and female participants, respectively, had laboratory-confirmed STIs, 19.7% of the female participants had endogenous infections. To the best of our knowledge, multiple previous studies have emphasized on women SRH, and have shown that the prevalence of RTIs in women varies from 18.5% to as high as 64.0% [19,20,21,22,23]. Only a few population-based studies on male SRH have revealed that 6.1–36.2% of the population are positive for STIs [12,13,24]. These variations might be attributed to factors such as different study profiles, and diagnosis criteria (whether it was self-reported or clinical diagnosis based on laboratory examination). A careful speculum and bimanual examination, a genital secretion test, and a blood test for both men and women can help identify multiple silent STIs/RTIs. After adjusting for age, the standardized rate of STIs was highest in Shanghai, which might be due to the population mobility and occupation distribution. Shanghai, is a city with the most mobile population and accounts for 4.3% of China’s internal migrant population. As previous studies have shown, risky sexual behaviors such as multiple partners, casual partners, and no condom use during the most recent sexual intercourse with the regular partner, were all significantly higher among the nonresidents compared to the residents in both men and women [25]. The univariable analysis showed that the highest prevalence of STIs was among laborers, who accounted for the majority of the internal migrants in Shanghai. Shendre et al. have also reported higher chances of developing STDs in unskilled workers [26].

Urumchi had the highest standardized rate of endogenous infections, which is probably due to poor hygienic habits and occupations. In Urumchi, while 95.8% of the subjects changed their underwear once in more than two days, 64.9% of them cleaned their genitals once in more than every two days, and 15.4% of them indulged in sexual behavior during menstruation. Moreover, in this study, the majority of Urumchi participants were engaged in service work, which is a risk factor for endogenous infections.

Ureaplasma, one of the most prevalent genotypes of mycoplasma, was the most common causative pathogen in both men and women with UU. Mycoplasma is an opportunistic pathogen that always coexists with host asymptomatically. UU, during pregnancy (16–20 weeks of gestational age), combined with other factors such as BV or cervical incompetence, often leads to premature rupture of membrane (PROM), preterm birth, stillbirth or fetal death, low birth weight, congenital malformations, and mycoplasmal pneumonia especially in preterm neonates [27,28]. Besides, UU is also associated with the male reproductive system and is more prevalent in infertile men than in fertile men [29]. An experimental study on rats has indicated that UU affects the sperm morphology leading to its death [30], though other studies have ruled out any association between UU and adverse fertility outcomes [31,32].

Adequate knowledge of RTIs could encourage appropriate prevention measures [33] and decrease the incidence of low genital tract infections [23]. In this study, the overall RTI-related knowledge was quite poor, with 3.2% and 22.6% of the participants scoring 0 points on the knowledge of RTI identification and RTI prevention questionnaires, respectively. Health-related decisions and actions of the participants are affected by their attitude towards preventing health problems [34]. Unwillingness to take RTI-related educational courses; therefore, impedes their chance to get appropriate information on reproductive health, including transmission and symptoms of RTIs, and appropriate family planning methods, thereby increasing the likelihood of exposure to these risk factors. In this study, frequent cleaning of one’s genitals and bathing have been associated with a high prevalence of STIs. Although, STIs are mostly caused by pathogens via unsafe and unprotected sexual behavior with multiple sexual partners or disloyal partners, many studies have demonstrated that genital hygiene practices also correlate with higher risks of STIs [35,36,37]. Inappropriate cleaning methods, such as vaginal touching, use of feminine wipes and alkaline lotion for genital and reproductive tract inevitably increase the chances of infection. In our qualitative interviews, we found that the majority of study participants, especially women used antiseptic wipes to clean their genitals, and many of these wipes in the Chinese market are of substandard quality [38]. All these factors disrupt the vaginal ecological balance and induce dysbacteriosis. Correct and consistent use of condoms can greatly reduce the risks of both pregnancy and STIs, including HIV transmission [39]. Condoms form a barrier to prevent direct contact with body fluids [40] and is the only and most effective contraceptive method that helps prevent the spread of STIs [41]. Oral contraceptives present no protection against viral STIs and HIV, and yeast infections are more common in people taking pills [42]. Meanwhile, many previous studies on birth control methods and STIs have revealed that oral or injectable hormonal contraceptives facilitate the risk of acquiring certain STDs, such as chlamydia and vaginal candidiasis, but decrease the risk for bacterial vaginosis [43,44,45]. However, contrary to the above findings, we found that oral contraceptives have an opposite relationship with STIs and have an insignificant relationship with endogenous infections. This could be due to the inadequate statistical power for the small sample size of oral contraceptive users (67/3320). As for endogenous infections, the more children the participant had, the greater were the odds of her contracting these infections. More children indicated more pregnancies and deliveries. Pregnancy is accompanied by compromised immunity and a higher incidence of yeast infections [42]. Endogenous infections are usually associated with hypoimmunity [27,46] and are characterized by repeated episodes. Delivery often causes soft birth canal injury, which in turn leads to infection. Some of our findings are consistent with previous reports [22,47,48]. Specifically, significant differences have been seen in the prevalence of STIs and endogenous infections in different socio-demographic contexts. In multivariable analyses, while age and sex were associated with the occurrence of STIs, age, occupation, and family per capita monthly income correlated with endogenous infections. Compared to the older participants, resistance to infections and sex hormone levels (which helps in strengthening vaginal immunity) were much better among the younger participants [27]. Meanwhile, women were more vulnerable to infections due to biological and social factors [47,49]. In this study, the majority of service workers were recruited from sauna rooms, entertainment venues, and hotels. These people often had irregular routines, high-intensity jobs, and unhealthy working environments. On the other hand, participants with higher income levels were more eager and able to improve their quality of life, including cleaner surroundings, accessible health services [48], and maintaining physical and mental health.

In light of the differences in the prevalence of STIs and endogenous infections in the three cities and their correlation with K.A.P, local governments need to establish reproductive health policies to emphasize the importance of maintaining good SRH. Simultaneously, explanation/counselling from doctors, brochures, and lectures, which were found to be the most popular education methods, could be adopted by reproductive health programs to improve the utilization of basic public services and cultivate reproductive health consciousness and behaviors. Health education centers and primary medical care units should provide convenient access for diagnosis and treatment of RTIs in workplace and community to facilitate health service seeking behavior.

This was a large multi-centric population-based cross-sectional study of the internal migrant population, which highlights the status of RTIs and related K.A.P. in this population. Moreover, we analyzed the relationship between K.A.P and RTIs. The study is beneficial to the subsequent intervention programs. However, there are some limitations to this study. First, there was a shortage of more common K.A.P. information, like sexual experience, number of homosexual sexual partners, access to and past behaviors of seeking care for RTIs symptoms, which might generate a limited correlation between current RTIs-related K.A.P and RTIs. Second, due to the private and sensitive nature of sexual behavior and the number of sexual partners, its impact on RTIs might have possibly been underestimated. Third, detailed information on the cleaning materials used, especially for the genitals, were collected by qualitative interviews rather than a structured questionnaire. Fourth, this is a cross-sectional study that will inevitably impede the drawing of causal inference.

## 6. Conclusions

This study documents a moderately high prevalence of RTIs among the internal migrant population of reproductive-age in three cities in China. Our results show that the knowledge of RTI identification and prevention among this population is quite poor. Additionally, the majority of respondents believed it was essential to develop RTI surveillance and showed great enthusiasm for SRH education. We have also demonstrated a correlation between K.A.P and the prevalence of RTIs in this population, after adjusting for the socio-demographic variables. To the best of our knowledge, there are only a few studies that have evaluated the prevalence of STIs and endogenous infections in male and female internal migrants, to separately explore the relationship between STIs/endogenous infections and K.A.P in China. This study emphasizes the need for better RTI-related K.A.P to improve the reproductive health of the internal migrant population in China.

## Figures and Tables

**Table 1:** Socio-demographic characteristics of the study participants.

Variables	Yinchuan (*N* = 1124)		Urumchi (*N* = 1015)		Shanghai (*N* = 1181)		Total (*N* = 3320)			*p*-Value ^b^
	*n*	%	*n*	%	*n*	%	*n*	%		
Age (years)										<0.0001
15–24	170	15.1	105	10.3	49	4.2	324	9.8		
25–34	547	48.7	684	67.4	431	36.5	1662	50.1		
35–44	302	26.9	214	21.1	435	36.8	951	28.6		
45–49	105	9.3	12	1.2	266	22.5	383	11.5		
Sex										<0.0001
Male	372	33.1	393	38.7	586	49.6	1351	40.7		
Female	752	66.9	622	61.3	595	50.4	1969	59.3		
Educational level										
Elementary school or lower	186	16.6	64	6.3	124	10.5	374	11.3		<0.0001
Junior high school	505	44.9	318	31.3	584	49.5	1407	42.4		
High school	265	23.6	309	30.4	262	22.2	836	25.2		
College or higher	168	15.0	324	31.9	211	17.9	703	21.2		
Occupation										<0.0001
Laborer	295	26.3	232	22.9	796	67.4	1323	39.9		
Service worker ^a^	336	29.9	341	33.6	72	6.1	749	22.6		
White-collar worker	107	9.5	44	4.3	191	16.2	342	10.3		
Other	386	34.3	398	39.2	122	10.3	906	27.3		
Marital status										<0.0001
Married	1105	98.3	1014	99.9	1061	89.8	3180	95.8		
Unmarried	19	1.7	1	0.1	120	10.2	140	4.2		
Family per capita monthly income (yuan)									<0.0001	
<3000	560	49.8	144	14.2	89	7.5	793	23.9		
3000–4999	394	35.1	463	45.6	624	52.8	1481	44.6		
5000–6999	124	11.0	274	27.0	282	23.9	680	20.5		
7000 and above	46	4.1	134	13.2	186	15.8	366	11.0		
Type of household registration (hukou)									<0.0001	
Rural	785	69.8	761	75.0	991	83.9	2537	76.4		
Urban	339	30.2	254	25.0	190	16.1	783	23.6		
Duration of staying in current residence per year (month)									<0.0001	
<6	98	8.7	14	34.0	34	2.9	146	4.4		
6~12	62	5.5	13	547.0	547	46.3	622	18.7		
>12	964	85.8	988	600.0	600	50.8	2552	76.9		

^a^ accommodation: 150/749, catering: 137/749, sauna: 221/749; entertainment: 241/749. ^b^ Indicates the application of the Cochran-Mantel-Haenszel (CMH) Chi-square test.

**Table 2:** RTI (reproductive tract infections)-related attitude and practices of the study participants.

Variables	Yinchuan (*N* = 1124)		Urumchi (*N* = 1015)		Shanghai (*N* = 1181)		Total (*N* = 3320)	
	*n*	%	*n*	%	*n*	%	*n*	%
Attitude								
Whether or not essential to develop RTIs census								
Yes	1058	94.1	859	84.6	887	75.1	2804	84.5
No	53	4.7	3	0.3	48	4.1	104	3.1
Uncertain	13	1.2	153	15.1	246	20.8	412	12.4
Whether or not willing to take RTI-related education courses								
Yes	1106	98.4	1011	99.6	1073	90.9	3190	96.1
No	18	1.6	4	0.4	108	9.1	130	3.9
Preferred education methods ^a^								
Explanation/counselling from doctors	884	78.6	498	49.1	635	53.8	2017	63.2
Brochures	921	81.9	538	53.0	435	36.8	1894	59.4
Lectures	865	77.0	218	21.5	170	14.4	1253	39.3
Books	843	75.0	208	20.5	137	11.6	1188	37.2
Newspapers	713	63.4	145	14.3	127	10.8	985	30.9
Broadcast/Television	470	41.8	332	32.7	189	16.0	991	31.1
Telephone Hotline	310	27.6	256	25.2	109	9.2	675	21.2
Posters	343	30.5	221	21.8	201	17.0	765	24.0
Others	110	9.8	48	4.7	79	6.7	237	7.4
Practice								
Personal hygiene								
Frequency of changing underwear								
Everyday	129	11.5	52	5.1	994	84.2	1175	35.4
Once in 2–3 days	785	69.8	556	54.8	156	13.2	1497	45.1
Once in ≥4 days	210	18.7	407	40.1	31	2.6	648	19.5
Frequency of cleaning genitals								
Everyday	196	17.4	401	39.5	1029	87.1	1626	49.0
Once in 2–3 days	684	60.9	476	46.9	129	10.9	1289	38.8
Once in ≥4 days	244	21.7	138	13.6	23	2.0	405	12.2
Frequency of taking bath								
Everyday	133	11.8	83	8.2	944	79.9	1160	34.9
Once in 2–3 days	590	52.5	329	32.4	206	17.4	1125	33.9
Once in ≥4 days	401	35.7	603	59.4	31	2.6	1035	31.2
Bath methods								
Shower	1013	90.1	987	97.2	1001	84.8	3001	90.4
Tub bath	57	5.1	26	2.6	116	9.8	199	6.0
Other	54	4.8	2	0.2	64	5.4	120	3.6
Whether or not having sexual behavior during menstruation								
Yes	287	25.5	190	18.7	182	15.4	659	19.8
No	837	74.5	825	81.3	999	84.6	2661	80.2
Whether or not having RTI symptoms in the last month								
Yes	313	27.9	233	23.0	280	23.7	826	24.9
No	811	72.2	782	77.0	901	76.3	2494	72.1
RTI symptoms ^b^								
Excessive or smelly genital secretion	260	23.1	232	22.9	198	16.8	690	83.5
Genital itching	148	13.2	148	14.6	154	13.0	450	54.5
Genital vesicle	28	2.5	1	0.1	87	7.4	116	14.0
Genital excrescence	30	2.7	2	0.2	86	7.3	118	14.3
Pain on urination	75	6.7	27	2.7	123	10.4	225	27.2
Contraceptive methods in use								
No	210	18.7	465	45.8	255	21.6	930	28.0
IUD (intrauterine devices)	236	21.0	206	20.3	267	22.6	709	21.4
Condom	476	42.4	252	24.8	406	34.4	1134	34.2
Pill	32	2.9	8	0.8	27	2.3	67	2.0
Sterilization	80	7.1	5	0.5	158	13.4	243	7.3
Others	90	8.0	79	7.8	68	5.8	237	7.1
Number of sexual partners								
Refusal	0	0.3	1	0.1	30	2.5	34	1.0
1	1116	99.3	1010	99.5	1113	94.2	3239	97.6
2	2	0.2	4	0.4	27	2.3	33	0.4
≥3	3	0.3	0	0.0	11	0.9	14	1.0
Number of children ^c^								
0	173	23.0	328	52.7	83	14.0	584	29.7
1	348	46.3	222	35.7	201	33.8	771	39.2
2	207	27.5	57	9.2	256	43.0	520	26.4
≥3	24	3.2	15	2.4	55	9.2	94	4.8

^a^ It is a multiple-choice question, and only participants with willingness to take RTI-related education courses answered it. ^b^ It is a multiple-choice question, and only participants with RTI symptoms in the last month answered it. ^c^ Only female participants answered it.

**Table 3:** RTI-related attitude and practices of the study participants in four age groups

Variables	15–24 years old (*N* = 324)		25–34 years old (*N* = 1662)		35–44 years old (*N* = 951)		45–49 years old (*N* = 383)		*p*-Value ^c^
	*n*	%	*n*	%	*n*	%	*n*	%	
Attitude									
Whether or not essential to develop RTIs census									0.8586
Yes	262	80.9	1419	85.4	806	84.8	317	82.8	
No	17	5.2	47	2.8	28	2.9	12	3.1	
Uncertain	45	13.9	196	11.8	117	12.3	54	14.1	
Whether or not willing to take RTI-related education courses									<0.0001
Yes	317	97.8	1624	97.7	898	94.4	351	91.6	
No	7	2.2	38	2.3	53	5.6	32	8.4	
Preferred education methods ^a^									
Explanation/counselling from doctors	191	59.0	989	59.5	601	63.2	236	61.6	0.0228
Brochures	185	57.1	987	59.4	537	56.5	185	48.3	0.0122
Lectures	135	41.7	685	41.2	313	32.9	120	31.3	<0.0001
Books	140	43.2	631	38.0	322	33.9	95	24.8	<0.0001
Newspapers	107	33.0	493	29.7	280	29.4	105	27.4	0.4559
Broadcast / Television	124	38.3	524	31.5	270	28.4	73	19.1	<0.0001
Telephone Hotline	62	19.1	384	23.1	176	18.5	53	13.8	0.0037
Posters	55	17.0	390	23.5	244	25.7	76	19.8	0.0189
Others	16	4.9	105	6.3	78	8.2	38	9.9	0.0189
Practice									
Personal hygiene									
Frequency of changing underwear									<0.0001
Everyday	57	17.6	435	26.2	442	46.5	241	62.9	
Once in 2–3 days	180	55.6	862	51.9	360	37.9	95	24.8	
Once in ≥4 days	87	26.9	365	21.9	149	15.7	47	12.3	
Frequency of cleaning genitals									<0.0001
Everyday	106	32.7	721	43.4	540	56.8	259	67.6	
Once in 2–3 days	165	50.9	716	43.1	324	34.1	84	21.9	
Once in ≥4 days	53	16.4	225	13.5	87	9.2	40	10.4	
Frequency of taking bath									<0.0001
Everyday	77	23.8	450	27.1	407	42.8	226	59.0	
Once in 2–3 days	132	40.7	618	37.2	276	29.0	99	25.9	
Once in ≥4 days	115	35.5	594	35.7	268	28.2	58	15.1	
Bath methods									<0.0001
Shower	288	88.9	1564	94.1	830	87.3	319	83.3	
Tub bath	16	4.9	71	4.3	68	7.2	44	11.5	
Other	20	6.2	27	1.6	53	5.6	20	5.2	
Whether or not having sexual behavior during menstruation									0.8108
Yes	79	24.4	312	18.8	185	19.5	83	21.7	
No	245	75.6	1350	81.2	766	80.6	300	78.3	
Whether or not having RTI symptoms in the last month									0.0421
Yes	64	19.8	408	24.6	256	26.9	98	25.6	
No	260	80.3	1254	75.4	695	73.1	285	74.4	
Contraceptive methods in use									<0.0001
No	140	43.2	504	30.3	198	20.8	88	23.0	
IUD	37	11.4	297	17.9	279	29.3	96	25.1	
Condom	117	36.1	654	39.4	300	31.6	63	16.5	
Pill	5	1.5	29	1.7	25	2.6	8	2.1	
Sterilization	2	0.6	50	3.0	99	10.4	92	24.0	
Others	23	7.1	128	7.7	50	5.3	36	9.4	
Number of sexual partners									0.0171
Refusal	2	0.9	4	0.6	12	1.3	9	2.4	
1	316	97.5	1614	98.7	923	97.1	359	93.7	
2	3	0.9	9	0.5	6	1.4	8	2.1	
≥3	2	0.6	2	0.1	3	0.3	7	1.8	
Number of children ^b^									0.0029
0	130	61.0	348	35.2	80	14.2	26	12.8	
1	66	31.0	433	43.8	219	38.8	53	26.1	
2	17	8.0	189	19.1	219	38.8	95	46.8	
≥3	0	0.0	18	1.8	47	8.3	29	14.3	

^a^ It is a multiple-choice question, and only participants with willingness to take RTI-related education courses answered it. ^b^ Only female participants answered it. ^c^ Indicates the application of the CMH Chi-square test.

**Table 4:** Distribution of laboratory-confirmed RTIs among three cities.

Variables	Yinchuan				Urumchi				Shanghai			
	Male (%)	Female (%)	*p*-Value	Total (%)	Male (%)	Female (%)	*p*-Value	Total (%)	Male (%)	Female (%)	*p*-Value	Total (%)
Bacterial vaginosis (BV)	-	16.4	-	-	-	17.4	-	-	-	15.0	-	-
Vulvovaginal candidiasis (VVC)	-	5.3	-	-	-	3.7	-	-	-	3.9	-	-
Endogenous infections	-	20.4	-	20.4	-	21.1	-	21.0	-	17.5	-	19.7
Trichomonal vaginitis (TV)	-	0.1	-	-	-	3.1	-	-	-	1.2	-	-
Chlamydia trachomatis (CT)	4.3	4.5	0.8663	4.5	5.3	5.5	0.9330	5.4	7.0	7.7	0.6292	7.4
Neisseria gonorrhoeae (NGH)	0.0	0.3	0.3197	0.2	0.0	0.2	0.4267	0.1	0.2	0.2	0.9914	0.2
Ureaplasma urealyticum (UU)	39.3	37.0	0.4584	37.7	38.4	48.9	0.0011	44.8	44.5	62.2	<0.0001	53.4
Human immunodeficiency virus (HIV)	0.0	0.0	-	0.0	0.0	0.0	-	0.0	0.2	0.0	-	0.1
Syphilis	0.5	0.4	0.7424	0.4	2.8	0.8	0.0130	1.6	0.9	0.2	0.0979	0.5
Sexually transmitted infections (STIs)	39.8	39.9	0.9721	39.9	39.7	50.5	0.0008	46.3	49.7	65.7	<0.0001	48.2

**Table 5:** Univariable analyses of the correlation between socio-demographic characteristics and RTIs among the internal migrant population.

Variables	Sexually Transmitted Infections			Endogenous Infections (Women)		
	*n*	%	*p*-Value ^a^	*n*	%	*p*-Value ^a^
Socio-demographic characteristics						
Area						
Yinchuan	448	39.9	<0.0001	153	13.6	0.2491
Urumchi	470	46.3		131	12.9	
Shanghai	682	57.8		104	8.8	
Age ^b^						
15–24	147	45.4	<0.0001	24	7.4	<0.0001
25–34	733	44.1		191	11.5	
35–44	483	50.8		124	13.0	
45–49	237	61.9		49	12.8	
Sex ^b^						
Male	595	44.0	<0.0001	-	-	-
Female	1005	51.0		-	-	
Educational level ^b^						
Elementary school or lower	199	53.2	<0.0001	76	20.3	0.0064
Junior high school	711	50.5		167	11.9	
High school	404	48.3		91	10.9	
College or higher	286	40.7		54	7.7	
Occupation ^b^						
Laborer	721	54.5	0.0006	114	8.6	<0.0001
Service worker	325	43.4		139	18.6	
White-collar worker	141	41.2		21	6.1	
Other	413	45.6		114	12.6	
Family per capita monthly income (yuan) ^b^						
<3000	330	41.6	0.0537	124	15.6	0.1528
3000–4999	729	49.2		166	11.2	
5000–6999	367	54.0		75	11.0	
7000 and above	174	47.5		23	6.3	
Type of household registration (hukou) ^b^						
Rural	1270	50.1	0.0067	298	11.8	0.4074
Urban	330	42.2		90	11.5	
RTI-related K.A.P (knowledge; attitude; and practices)						
Whether or not think it is essential to develop surveillance for RTI ^b^						
Yes	1303	46.5	0.0168	348	12.4	0.0272
No	55	52.9		1	1.0	
Uncertain	242	58.7		39	9.5	
Whether or not willing to take RTI-related education ^b^						
Yes	1515	47.5	0.0018	381	11.9	0.0256
No	85	65.4		7	5.4	
Frequency of cleaning genitals ^b^						
Everyday	901	55.4	0.0003	179	11.0	0.4513
Once in 2–3 days	547	42.4		168	13.0	
Once in ≥ 4 days	152	37.5		41	10.1	
Frequency of taking bath ^b^						
Everyday	669	57.7	0.0004	106	9.1	0.1068
Once in 2–3 days	508	45.2		142	12.6	
Once in ≥4 days	423	40.9		140	13.5	
Bath methods ^b^						
Shower	1431	47.7	0.2100	353	11.8	0.0951
Tub bath	113	56.8		28	14.1	
Other	56	46.7		7	5.8	
Whether or not having RTI symptoms in the last month ^b^						
Yes	449	54.4	<0.0001	222	32.9	<0.0001
No	1151	46.2		166	12.8	
Contraceptive methods ^b^						
None	472	50.8	<0.0001	88	9.5	0.0685
IUD	370	52.2		94	13.3	
Condom	473	41.7		116	10.2	
Pills	24	35.8		13	19.4	
Sterilization	147	60.5		38	15.6	
Other	114	48.1		39	16.5	
Number of children ^b^						
0	-	-	-	87	9.1	<0.0001
1	-	-		145	14.0	
2	-	-		131	21.0	
≥3	-	-		25	22.1	

Notation: Marital status, duration of stay in residence (months), the frequency of changing underwear, whether or not having sexual behavior during menstruation, and the number of sexual partners all showed no significant association with STIs and endogenous infections. ^a^ Indicates the application of the CMH Chi-square test. ^b^ Implicates the adjusted region effect.

**Table 6:** Multivariable analyses of the correlation between RTI-related K.A.P and prevalence of RTIs among the internal migrant population.

Variables		Sexually Transmitted Infections			Endogenous Infections (Female)		
Comparable G	Reference G	Odds Ratio (OR)	95% Confidence Interval (CI)	*p*-Value	OR	95% CI	*p*-Value
RTI-related K.A.P							
Attitude							
Whether or not willing to take RTI-related educational courses							
No	Yes	1.6	1.1–2.3	0.0201	0.4	0.2–0.9	0.0226
Practices							
Frequency of changing underwear							
Once in 2–3 days	Everyday	1.5	1.1–1.9	0.0058	-	-	-
Once in ≥4 days		1.8	1.3–2.6	0.0013	-	-	-
Frequency of cleaning genitals							
Once in 2–3 days	Everyday	0.8	0.6–0.9	0.0136	-	-	-
Once in ≥4 days		0.7	0.5–1.0	0.0567	-	-	-
Frequency of taking a bath							
Once in 2–3 days	Everyday	0.8	0.6–1.0	0.0272	-	-	-
Once in ≥4 days		0.5	0.4–0.7	<0.0001	-	-	-
Whether or not having RTI symptoms in the last month							
Yes	No	1.3	1.1–1.6	0.0008	3.2	2.5–4.0	<0.0001
Number of children							
1	0	-	-	-	1.2	0.9–1.7	0.2740
2		-	-	-	1.7	1.1–2.5	0.0112
≥3		-	-	-	1.8	1.0–3.3	0.0624
Contraceptive methods							
None	Sterilization	0.9	0.6–1.2	0.4621	-	-	-
IUD		0.97	0.6–1.20	0.3844	-	-	-
Condom		0.7	0.5–0.9	0.0161	-	-	-
Pills		0.4	0.2–0.8	0.0055	-	-	-
Others		0.7	0.5–1.1	0.1214	-	-	-

Notation: Socio-demographic characteristics, including area, age, sex, educational level, occupation, marital status, family per capita monthly income (yuan), type of household registration (hukou), and duration of stay in the current residence per year (months), were all controlled during multivariable analyses.

## Supplementary Materials

The following are available online at https://www.mdpi.com/1660-4601/16/4/655/s1, Table S1: Socio-demographic characteristics variables, Table S2: RTI-related knowledge, attitude, and practice variables, Table S3: Laboratory-confirmed RTIs species, Table S4: Internal migrant population of reproductive age from the Sixth Nationwide Population Census.
