# Supplementary material for: The Prevalence of Reproductive Tract Infections in a Chinese Internal Migrant Population, and Its Correlation with Knowledge, Attitude, and Practices: A Cross-Sectional Study
Source: Int J Environ Res Public Health. 2019 Feb 22;16(4):655. doi: 10.3390/ijerph16040655 (PMC6406905; doi:10.3390/ijerph16040655)
Supplement: Supplementary file 1 [file ijerph-16-00655-s001.pdf]

International Journal of
Environmental Research and Public Health

MDPI

Supplementary appendix

The Prevalence of Reproductive Tract Infections in a Chinese Internal Migrant Population, and Its Correlation with Knowledge, Attitude, and Practices: A Cross-Sectional Study

Shuangfei Xu [1,2,3], Chuanning Yu [4], Ying Zhou [1,2], Junqing Wu [1,2], Tieling Bai [5], Junxian Zhang [6], Yuyan Li [1,2,*]

International Journal of
Environmental Research and Public Health

MDPI

Table S1. Socio-Demographics Characteristics variables.

Variable	Category	Code	Scale
Area	Yinchuan	1	Nominal
	Urumchi	2	
	Shanghai	3	
Age	15-24	1	Ordinal
	25-34	2	
	35-44	3	
	45-49	4	
Sex	Male	1	Dichotomous
	Female	2	
Educational level	Elementary school or lower	1	Ordinal
	Junior high school	2	
	High school	3	
	College or higher	4	
Occupation	Laborer	1	Nominal
	Service worker	2	
	White-collar worker	3	
	Other (unemployed or self-employed)	4	
Marital status	Married	1	Dichotomous
	Unmarried	2	
Family per capita monthly income (CNY)	<1000	1	Ordinal
	1000-2999	2	
	3000-4999	3	
	5000-6999	4	
	>7000	5	
Type of household registration (hukou)	Rural	1	Dichotomous
	Urban	2	
Duration of staying in residence per` year (month)	<6	1	Ordinal
	6-	2	
	12	3	

International Journal of
Environmental Research and Public Health

MDPI

Table S2. RTIs-Related Knowledge, Attitude and Practice variables.

Variable	Category	Code	Scale
**Knowledge**			
RTIs identification knowledge			Continuous
RTIs prevention knowledge			Continuous
**Attitude**			
Whether or not essential to develop RTIs census	Yes	1	Nominal
	No	2	
	Uncertain	3	
Whether or not willing to take RTIs related education courses	Yes	1	Dichotomous
	No	2	
Preferred education methods a			
explanation/counselling from doctors	Yes	1	Dichotomous
	No	2	
Brochures	Yes	1	Dichotomous
	No	2	
Lectures	Yes	1	Dichotomous
	No	2	
Books	Yes	1	Dichotomous
	No	2	
Newspapers	Yes	1	Dichotomous
	No	2	
Broadcast / Television	Yes	1	Dichotomous
	No	2	
Telephone Hotline	Yes	1	Dichotomous
	No	2	
Posters	Yes	1	Dichotomous
	No	2	
Others	Yes	1	Dichotomous
	No	2	
**Practice**			
Personal hygiene			
Frequency of changing underwear	Everyday	1	Ordinal
	Once in 2-3 days	2	
	Once in ≥ 4 days	3	
Frequency of cleaning genitals	Everyday	1	Ordinal
	Once in 2-3 days	2	
	Once in ≥ 4 days	3	
Frequency of taking bath	Everyday	1	Ordinal
	Once in 2-3 days	2	
	Once in ≥ 4 days	3	
Bath methods	Shower	1	Nominal
	Tub bath	2	
	Other	3	
Whether or not having sexual behavior during menstruation	Yes	1	Dichotomous
	No	2	
Whether or not having RTI symptoms in last month	Yes	1	Dichotomous
	No	2	
RTI symptoms			

excessive or smelly genital secretion	Yes	1	Dichotomous
	No	2	
genital itching	Yes	1	Dichotomous
	No	2	
genital vesicle	Yes	1	Dichotomous
	No	2	
genital excrescence	Yes	1	Dichotomous
	No	2	
pain on urination	Yes	1	Dichotomous
	No	2	
Contraceptive methods in use	No	1	Nominal
	IUD	2	
	Condom	3	
	Pill	4	
	Sterilization	5	
	Others	6	
Number of sexual partners	Refusal	1	
	1	2	
	2	3	
	≥3	4	
Number of children	0	1	Ordinal
	1	2	
	2	3	
	≥3	4	

International Journal of
Environmental Research
and Public Health

MDPI

Table S3. Laboratory- confirmed RTIs species.

Variable	Category	Code	Scale
Sexually transmitted infections (STIs)			
Trichomonal vaginitis (TV)	Yes	1	Dichotomous
	No	2	
Chlamydia trachomatis (CT)	Yes	1	Dichotomous
	No	2	
Neisseria gonorrhoeae (NGH)	Yes	1	Dichotomous
	No	2	
Ureaplasma urealyticum (UU)	Yes	1	Dichotomous
	No	2	
Syphilis	Yes	1	Dichotomous
	No	2	
Human immunodeficiency virus (HIV)	Yes	1	Dichotomous
	No	2	
Endogenous Infections			
Bacterial vaginosis (BV)	Yes	1	Dichotomous
	No	2	
vulvovaginal candidiasis (VVC)	Yes	1	Dichotomous
	No	2	

International Journal of
Environmental Research and Public Health

MDPI

Table S4. Internal migrant population of reproductive age from the Sixth Nationwide Population Census.

	Male		Female		Total
	**n**	**%**	**n**	**%**	
Age					
15-24	12393873	53.30	10857514	46.70	23251387
25-34	13312862	56.16	10392491	43.84	23705353
35-44	11741181	58.12	8461880	41.88	20203061
45-49	3557300	60.41	2330888	39.59	5888188

International Journal of Environmental Research and Public Health

MDPI

QUESTIONNAIRE

Socio-demographic information

A1 Birthday _________ (yyyymmdd)

A2 Gender

① Male ②Female

A3 Educational level

① Elementary school or lower ② Junior high school

③ High school ④ College or higher

A4 Occupation

① Laborer ② Service worker

③ White-collar worker ④ Other (unemployed or self-employed)

A5 Marital status

① Married ② Unmarried

A6 Family per capita monthly income (CNY)

① <1000 ② 1000-2999 ③ 3000-4999

④ 5000-6999 ⑤ >7000

A7 Type of household registration (hukou)

① Rural ② Urban

A8 Duration of staying in residence per year (month)

① <6 ② 6- ③ 12

Knowledge

A9 The score of RTIs identification knowledge

A10 The score of RTIs prevention knowledge

Attitud

A11 Whether or not essential to develop RTIs census

① Yes ② No ③Uncertain

A12 Whether or not willing to take RTIs related education courses

① Yes ② No (**JUMP TO A13**)

Prederren on education methods (multiple choice questions):

A12A ① explanation/counselling from doctors A12B ② Brochures

A12C ③ Lectures A12D ④ Books A12E⑤ Newspapers

A12F ⑥ Broadcast / Television A12G⑦ Telephone Hotline

A12H ⑧ Posters A12I ⑨ Others____________

Practice

Personal hygiene

A13 Frequency of changing underwear

① Everyday ② Once in 2-3 days ③ Once in ④ ≥ 4 days

A14 Frequency of cleaning genitals

① Everyday ② Once in 2-3 days ③ Once in ④ ≥ 4 days

A15 Frequency of taking bath

① Everyday ② Once in 2-3 days ③ Once in ④ ≥ 4 days

International Journal of Environmental Research and Public Health

MDPI

A16 Bath methods

① Shower ② Tub bath ③ Other

A17 Whether or not having sexual behavior during menstruation

① Yes ② No

A18 Number of children

① 0 ② 1 ③ 2 ④ ≥ 3

A19 Whether or not having RTI symptoms in last month

① Yes ② No (**JUMP TO A20**)

RTI symptoms in last month (multiple choice questions):

A19A ① excessive or smelly genital secretion A19B ② genital itching

A19C ③ genital vesicle A19D ④genital excrescence A19E ⑤pain on urination

A20 Contraceptive methods in use

① No ② IUD ③ Condom ④ Pill ⑤ Sterilization ⑥ Others

A21 Number of sexual partners

① 0 ② 1 ③ 2 ④ ≥ 3

INVESTIGATOR_______________

DATA___________(YYYYMMDD)
